# *Coronaviridae* and SARS-associated Coronavirus Strain HSR1

**DOI:** 10.3201/eid1003.030683

**Published:** 2004-03

**Authors:** Elisa Vicenzi, Filippo Canducci, Debora Pinna, Nicasio Mancini, Silvia Carletti, Adriano Lazzarin, Claudio Bordignon, Guido Poli, Massimo Clementi

**Affiliations:** * San Raffaele Scientific Institute, Milan, Italy; † University “Vita-Salute” San Raffaele, Milan, Italy

**Keywords:** SARS-coronavirus, isolation, sequence, plaque assay, real-time PCR

## Abstract

During the recent severe acute respiratory (SARS) outbreak, the etiologic agent was identified as a new coronavirus (CoV). We have isolated a SARS-associated CoV (SARS-CoV) strain by injecting Vero cells with a sputum specimen from an Italian patient affected by a severe pneumonia; the patient traveled from Vietnam to Italy in March 2003. Ultrastructural analysis of infected Vero cells showed the virions within cell vesicles and around the cell membrane. The full-length viral genome sequence was similar to those derived from the Hong-Kong Hotel M isolate. By using both real-time reverse transcription–polymerase chain reaction TaqMan assay and an infectivity plaque assay, we determined that approximately 360 viral genomes were required to generate a PFU. In addition, heparin (100 μg/mL) inhibited infection of Vero cells by 50%. Overall, the molecular and biologic characteristics of the strain HSR1 provide evidence that SARS-CoV forms a fourth genetic coronavirus group with distinct genomic and biologic features.

An outbreak of atypical pneumonia, referred to as severe acute respiratory syndrome (SARS), was identified in the Guangdong Province of People’s Republic of China at the end of 2002 and spread to other Asian countries and Canada (*1*,*2*) from February through March 2003. Individual cases (all in persons infected in Asia) were diagnosed in Europe during the same period (*3*). A novel human coronavirus (SARS-associated coronavirus [SARS-CoV]) has been isolated from the oropharyngeal specimens of patients with SARS (*4*,*5*). Experimental infection of macaques has confirmed that the SARS-CoV is the cause of SARS (*6*,*7*).

Coronaviruses are enveloped, positive-stranded RNA viruses associated with enteric and respiratory diseases in animals and humans; they are currently classified into three antigenic groups: group 1 and 2 include mammalian coronaviruses, and group 3 encompasses avian coronaviruses. Human coronaviruses are associated with common cold-like diseases and are included in both group 1 (CoV-229E) and 2 (CoV-OC43) (*8*). Sequence analysis of the complete genome of SARS-CoV has shown an RNA molecule of about 29,750 bases in length, with a genome organization similar to that of other coronaviruses (*9*–*11*). In spite of this similar organization, the SARS-CoV RNA sequence is only distantly related to that of previously characterized coronaviruses (*9*). Consequently, whether the SARS-CoV has “jumped” from a nonhuman host reservoir to humans and the molecular basis of such a jump remain unanswered questions (*12*). Some biologic features of the SARS-CoV described in vivo and in vitro differ from those of other coronaviruses previously identified. Among these features are the peculiar tropism of the virus for Vero cells (a continuous cell line established from monkey kidney epithelial cells), its capacity for growth at 37°C (while other respiratory coronaviruses grow at lower temperatures), and its ability to infect lower respiratory tract tissues (*13*). These aspects render the molecular and biologic characterization of SARS-CoV important not only for understanding the determinants of its pathogenic potential but also for planning rational strategies of antiviral therapy and vaccination.

We have recently obtained a SARS-CoV isolate from a frozen sputum sample collected from an Italian patient affected by a respiratory disease of unknown cause; onset of illness began during the patient’s travel to Vietnam in March 2003. The viral strain has been designated as SARS-CoV HSR1. To gain insight into SARS-CoV biopathology, we analyzed the relevant features of SARS-CoV HSR1 growth in vitro, including the ultrastructural analysis of the consequences of virus replication in Vero cells. We have optimized both a reverse transcription–polymerase chain reaction (RT-PCR) TaqMan assay for quantifying the number of viral genomes and a plaque assay for performing titration of the virus infectivity. In addition, we have completely sequenced the viral genome of the SARS-CoV HSR1 and compared it to other SARS-CoV strains recently isolated in disease-epidemic areas.

## Methods

### SARS-CoV Isolation

In March 2003, an Italian man who recently traveled from Vietnam to Italy was affected by a respiratory disease of unknown cause; he was hospitalized in a clinical unit for acute infectious diseases in a public hospital in Milan, Italy. A sputum sample was collected at the peak of illness and stored at –80°C. Isolation of SARS-CoV was performed with Vero cells maintained in Dulbecco’s modified Eagle medium (D-MEM, BioWhittaker, Verviers, Belgium) and supplemented with 10% fetal calf serum (FCS, HyClone, Perbio Science Erembodegem-Aalst, Belgium), penicillin/streptomycin (BioWhittaker), and 2.5 μg/mL Fungizone (Invitrogen Ltd, Life Technologies, Paisley, UK) (complete medium). In detail, an aliquot (0.5 mL) of the sputum sample was mixed with 2 x 10^6^ Vero cell suspension in 2 mL of complete medium. After incubation for 1 h at 37°C, 5% CO_2_, 4 mL of complete medium was added, and the cell suspension was transferred into a 25-cm^2^ tissue culture flask (Falcon, Becton Dickinson Labware, Lincoln Park, NJ). Twenty-four hours after inoculation, the cell cultures were examined with an optical microscope for evidence of cytopathic effects (CPE). An aliquot of culture supernatant was collected and stored at –80°C for RT-PCR evaluation. After an additional 24 hours, both the culture supernatant and the cells were collected after 2 cycles of freeze thawing followed by clarification of the thawed contents by centrifugation at 1,000 x *g* and dispensation of the supernatant into aliquots stored at –80°C (primary viral stock). For generation of the secondary and tertiary viral stocks, adherent Vero cells flasks were seeded in a 25 cm^2^ tissue culture and injected with 0.5 mL of stored supernatant filtered with 0.45 μ filters. Three days after infection, the cells and supernatant were subjected to 2 cycles of freeze thawing, and the supernatant was collected after centrifugation followed by filtration as described above (secondary viral stock). Finally, Vero cells seeded in 2 flasks of 75 cm^2^ were injected with 1.5 mL of the second passage virus stock in a total volume of 25 mL to generate a third-passage viral stock.

### RT-PCR Amplification of Viral Sequences

Direct SARS-CoV RNA amplification was performed starting from the sputum sample treated with an equal volume of phosphate-buffered solution (PBS). RNA was purified from 750 µL of the resulting homogenate by using the Qiagen Viral RNA Mini Kit (Qiagen, Inc., Santa Clarita, CA) (elution volume 50 μL), according to manufacturer’s instructions. Viral RNA was extracted from 0.5 mL of culture supernatant with the same kit (elution volume 50 μL). cDNA synthesis and subsequent amplification were performed by using either nested RT-PCR or real-time RT-PCR approach, as described elsewhere, with minor modifications (*3*). In brief, 5 μL of extracted RNA was reverse-transcribed for 30 min at 42°C by using Mo-MuLV RT, a mixture of random hexamers, and an oligo-(dT) primer. Two microliters of the synthesized cDNA was subsequently amplified by using the following primers pairs: outer primers: BNI-OUTS2, 5′-ATG AAT TAC CAA GTC AAT GGT TAC-3′; BNI-OUTAS, 5′-CAT AAC CAG TCG GTA CAG CTA C-3′; inner primers: BNI-INS, 5′-GAA GCT ATT CGT CAC GTT CG,-3′; BNI­INAS, 5′-CTG TAG AAA ATC CTA GCT GGA G-3′. A sequence of the SARS-CoV RNA encompassing the target region was used as positive control, and a sputum sample from a SARS-negative healthy donor and a feline coronavirus RNA extract were used as negative controls. The amplified PCR product was separated by agarose gel electrophoresis visualized by ethidium bromide staining and sequenced directly on an ABI PRISM 3100 Genetic Analyzer with ABI PRISM BigDye Terminator v3.0 sequencing kit (Applied Biosystems, Foster City, CA). SARS-CoV was then identified by searching for homologies between the amplified fragment and previously deposited sequences using BLAST. Finally, the quantitation of the viral RNA was performed in a TaqMan assay after generation of cDNA. The primer pair and probe BNITMSARS1, 5′-TTATCACCCGCGAAGAAGCT-3′; BNITMSARAS2, 5′-CTCTAGTTGCATGACAGCCCTC-3′, BNI-TMSARP 6-carboxifluorescein-TCG TGC GTG GAT TGG CTT TGA TGT-6 carboxy-tetramethylrhodamin (*3*) were added to the universal PCR master mix (Applied Biosystems) at 200 and 120 nM, respectively, in a final volume of 25 μL. The standard was obtained by cloning the 77-bp fragment into the pCR2.1 plasmid by using the TA cloning kit (InVitrogen Corp., San Diego, CA). A linear distribution (r = 0.99) was obtained between 10^1^ to 10^8^ copies.

### SARS-CoV Plaque Infectivity Assay

To have the most effective quantitative assay of virus infectivity and to compare the infectious titer with quantitative molecular assays, we optimized a plaque assay (determining the PFU/mL). Confluent Vero cells in 6 well plates (Falcon) were incubated in duplicate with 1 mL of PBS containing 100 μL of SARS-CoV HSR1 viral stock in 10-fold serial dilutions from –10e2 (1/100) to –10e8 (1/100,000,000). After 1 hour of incubation, the viral inoculum was removed and 1 mL of 1% carboxymethylcellulose (Sigma Chemical Corp., St. Louis, MO) overlay with DMEM supplemented with 1% fetal calf serum was added to each well. After 6 days of incubation, the cells were stained with 1% crystal violet (Sigma) in 70% methanol. The plaques were counted after being examined with a stereoscopic microscope (SMZ-1500, Nikon). The virus titer was calculated in PFU per milliliter.

### Ultrastructural Analysis

Adherent Vero cells were infected with 5 x 10^4^ PFU/mL of SARS-CoV HSR1 at the third passage in a T-75 flask. Twenty-four hours after infection, Vero cells were detached from the tissue culture flask with a cell scraper, and the cell suspension was centrifuged at 173 x *g* for 4 min. The pellet was resuspended in PBS without Ca^++^ and Mg^++^, and the suspension was spun at 173 x *g* for an additional 4 min. The cell pellet was resuspended and fixed in 4% formaldehyde and 2.5% glutaraldehyde in cacodylate buffer and incubated for 5 min at room temperature. The sample was then centrifuged at 13,414 x *g* for 5 min; the pellet was fixed with 2% OsO_4_ in 2.5% glutaraldehyde in cacodylate buffer for 60 minutes. The pellet was dehydrated in graded ethanol, washed in propylene oxide and infiltrated for 12 hours in a 1:1 mixture of propylene oxide and epoxidic resin (Epon). Cells were then embedded in Epon and polymerized for 24 hours at 60°C. Slides were cut with ultramicrotome (Ultracut Uct, Leica, Deerfield, IL), stained with uranyl acetate and lead citrate, and metaled. The ultrathin sections of infected Vero cells were observed through transmission electron microscopy (Hitachi H7000).

### SARS-CoV HSR1 Genome RNA and Phylogenetic Reconstruction

Sequencing of the complete SARS-CoV HSR1 genome was performed by using 68 partially overlapping primers encompassing the whole viral genome. A 5′ rapid amplification of PCR ends (RACE) technique was performed to capture the 5′-untranslated region of the genome (10). Each 750-bp fragment was gel-isolated by means of a QIAQuick gel Extraction kit (Qiagen) and directly sequenced from both directions inward and outward. SeqScape version 2.0 (Applied Biosystems) software was used for base calling, editing, and assembly of the fragments. Manual check of the differences between the electropherograms and the reference sequence (Urbani isolate) was performed and eventually led to reamplification or resequencing of some fragments. The complete sequence of SARS-CoV HSR1 strain was aligned with all the previously sequenced full-length genomes available from the GenBank database by using ClustalW and editing with BioEdit version 5.0.9 for manual corrections. Available full-length sequences of SARS-CoV strains accessed from GenBank and used for phylogenetic analysis and genotyping were as follows: Taiwan TC2 (AY338175), Taiwan TC1 (AY338174), TWC (AY321118), Sin2774 (AY283798), Sin2748 (AY283797), Sin2679 (AY283796), Sin2677 (AY283795), Sin2500 (AY283794), Frankfurt 1 (AY291315), BJ04 (AY279354), BJ03 (AY278490), BJ02 (AY278487), GZ01 (AY278489), CUHK-W1 (AY278554), ZJ01 (AY297028), TOR2 (AY274119), TW1 (AY291451), CUHK­Su10 (AY282752), BJ01 (AY278488), Urbani (AY278741), and HKU-39849 (AY278491). Sequences were trimmed to equivalent length and phylogenetic relationships were estimated with PAUP* (maximum-parsimony and maximum-likelihood methods by using the p-distance model) and MEGA version 2.1. Trees were edited by using Treeview.

## Results

**Figure 1:**
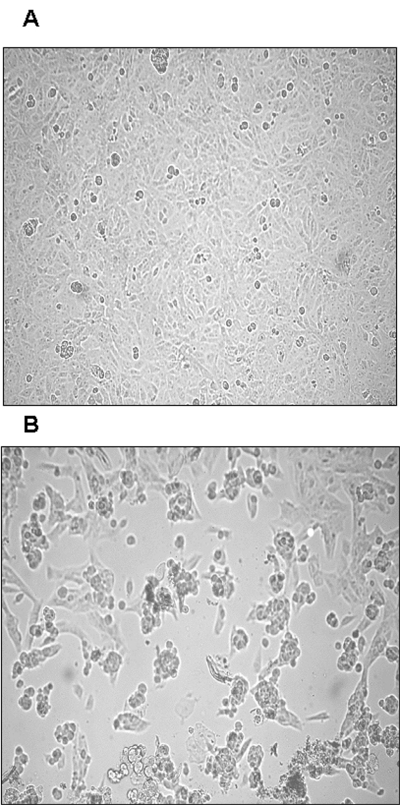
Cytopathic effect (CPE) of primary severe acute respiratory syndrome–associated coronavirus strain HSR1 isolate. A**,** uninfected Vero cells form a continuous monolayer of spindle-shaped cells. B, a strong CPE was observed after 24 hours of incubation of Vero cells with the patient sputum sample (primary isolate).

**Figure 2:**
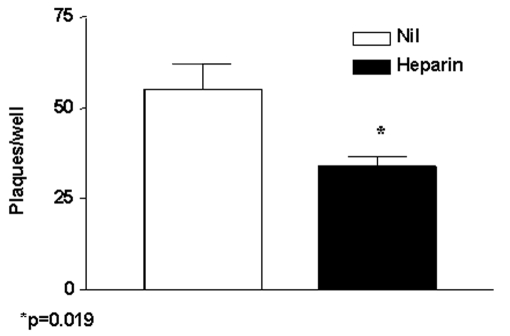
Effect of the sulfated polysaccharide heparin (100 μg/mL) added 30 minutes before injecting Vero cells with 100 PFU/mL of severe acute respiratory syndrome–associated coronavirus strain HSR1. The results are expressed as number of plaques/well and represent the mean ± SEM of two independent experiments each carried out in duplicate cultures. The p value was calculated by the Mann-Whitney U test.

Both nested RT-PCR and the real-time RT-PCR assays performed on the sputum sample from a person with SARS tested positive for SARS-CoV RNA. The viral load in the sample was estimated to be 5.6 x 10^4^ SARS-CoV RNA copies/mL. Twenty-four hours after injection of Vero cells with the sputum sample, a strong CPE was observed, as indicated in Figure 1B. The CPE was diffused with cell rounding with refractive appearance, and the cell monolayer was destroyed as compared to control uninfected Vero cells (Figure 1A). The viral load in the culture supernatant 24 hours after injection with the clinical sample was 1.3 x 10^5^ copies/mL as determined by quantitative real-time RT-PCR. Serial passage of the virus on Vero cells to obtain a tertiary viral stock yielded 9.1 x 10^8^ copies/mL. A plaque assay was optimized to determine the in vitro infectivity of SARS-CoV. The tertiary viral stock tested 2.5 x 10^6^ PFU/mL in the plaque assay, suggesting that about 360 genomes were required to generate a single plaque in tissue cultures, at least under the conditions described here. We also used the plaque assay for testing the potential inhibitory effect on virus infectivity of a single concentration of heparin, the prototypic compound of a class of inhibitors of virus entry for enveloped viruses including HIV type 1 (HIV-1) (*14*) and herpes simplex virus 1 and 2 (HSV-1 and 2) (*15*). Heparin indeed reduced the formation of plaques by 50% when added 30 min before infection of Vero cells with 100 PFU/mL of the SARS-CoV (Figure 2).

**Figure 3:**
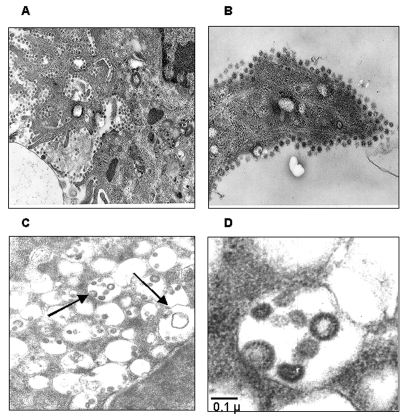
Ultrastructural analysis of Vero cells infected with severe acute respiratory syndrome–associated coronavirus (SARS-CoV) strain HSR1. A, intracellular budding of SARS-CoV in large vesicles containing CoV virions (magnification x30,000); B, clusters of extracellular virions adjacent to the plasma membrane (magnification x50,000); C and D, intracellular budding of SARS-CoV virions (magnification x50,000).

Thin-section electron microscopy showed the typical features of intracellular CoV particles. Cells were engulfed with viral particles localized in cytoplasmic vesicles (Figure 3A). This feature is typical of all *Coronaviridae* viruses that bud intracellularly at membranes of the intermediate compartment between the endoplasmic reticulum and the Golgi complex, whereas newly assembled virions reach the cell surface by vesicular transport (*16*). After the extracellular release, virus particles were found in large clusters adjacent to the plasma membrane, as evidenced in Figure 3B. Overall, the ultrastructural analysis documented that SARS-CoV cultivated in Vero cells behaved like a typical coronavirus, characterized by intracellular budding (Figure 3, panels C and D).

**Figure 4:**
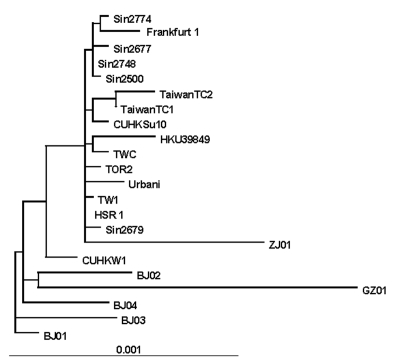
Phylogenetic tree obtained by applying PAUP* (maximum-likelihood methods using the p-distance model) applied to complete genome sequences of the severe acute respiratory syndrome–associated coronavirus (SARS-CoV) HSR1 strain and the 21 other SARS-CoV isolates.

The sequence of SARS-CoV strain HSR1 genomic RNA was 29,751 bases in length, with a polyanion tail. We identified the major open reading frames (ORFs), coding for the 4 major structural proteins, namely: spike (S), envelope (E) matrix (M), nucleocapsid (N) gene products, and at least other 10 proteins, including a few of unknown function. The complete sequence of SARS-CoV strain HSR1 has been deposited in the GenBank database (GenBank accession no. AY323977). This sequence was aligned with those of the other 21 SARS-CoV isolates to facilitate phylogenetic analysis. Overall, the mean difference in nucleotide composition between all the isolates was 18 ± 1 nt variations within the whole genomes, thus confirming the genetic conservation observed previously (*9*). On the whole, 149 sites were variable among the 22 aligned isolates, and 24 loci in the viral genome varied in more than one isolate, including in the SARS-CoV HSR1 genome (recurrent mutations). Eleven of 24 recurrent mutations were silent, whereas 3 of 13 mutations generating amino acid substitutions were observed within the N-terminal domain of the spike glycoprotein gene (positions 21,722, 22,223, and 22,423, determining a G to D, an I to T, and a G to R amino acid change, respectively). Six mutations were detected in the replicase gene (positions 8,572, 9,404, 9,479, 9,854, 17,564, and 19,084, determining a V to L, a V to A, a V to A, an A to V, a D to E, and a T to I amino acid change, respectively), and 1 mutation was found in the matrix protein (position 26,600, determining an A to V amino acid change). The phylogenetic analysis was also performed by using the maximum parsimony method, considering only sequence variants that recurred in more than one strain in order to reduce the mutational noise caused by PCR or sequencing mistakes. Figure 4 shows the phylogenetic tree obtained with the maximum likelihood method. The maximum parsimony trees produced the same structure with minor changes in the subtrees’ branch patterns (data not shown). In these analyses, SARS-CoV HSR1 appears to be strongly related to strains isolated from patients who had traveled from Hong Kong to different geographic areas (Singapore, Canada, Vietnam) and spread the infection to their home countries in a few cases.

## Discussion

The isolation of a novel CoV in persons with SARS and proof of its etiologic role in this disease underscore the importance of identifying the pathogenic determinants of SARS-CoV. This information may be central to plan specific therapeutic and preventive strategies against SARS. Major areas of research include the following: 1) analyzing growth characteristics of SARS-CoV in vitro; 2) evaluating virus-host relationships at the molecular level; 3) understanding both SARS-CoV genome organization and the role of nonstructural proteins of unknown function; 4) studying the evolutionary relationships of different coronaviruses of animal and human origin; and 5) identifying targets for anti-CoV chemotherapy and vaccination. In our study, we characterized a novel isolate of SARS-CoV (designated HSR1 strain). The analysis of the complete sequence of SARS-CoV HSR1 genome showed the same organization in ORFs previously described in this novel human virus (*10*) and confirmed the relative genetic stability among the different isolates (*9*). However, for other SARS-CoVs isolates, amino acid changes were observed in this HSR1 strain at the level of the N-terminal domain of the spike glycoprotein, the viral replicase, and the matrix protein. Despite this relative stability, phylogenetic analysis of the HSR1 isolate (Figure 4) has shown that the virus is more related to the strains isolated in patients who had traveled from Hong Kong to different geographic areas, such as Singapore, Canada, and Vietnam, than to the Beijing isolates. In addition, we have observed that other biomolecular features shared by most *Coronaviridae* coexist in SARS-CoV HSR1 with particular characteristics that seem to be unique of the novel virus. In particular, SARS-CoV is able to replicate in Vero cells with a rapid production of high virus titers and fast CPE. By using quantitative molecular and biologic assays, we could estimate that, under our experimental conditions, 360 genomes are approximately required to form a plaque of infectivity in vitro. SARS-CoV seems unlike most of the other respiratory coronaviruses infecting humans, which fail to grow efficiently in tissue cultures and are easily detectable by using PCR amplification methods only (*17*). Infection in Vero cells showed most of the usual characteristics of the CoV replication (*16*), including intracellular budding of virions, as demonstrated by electron microscopy.

To investigate whether SARS-CoV could be inhibited by polyanions, we tested the effects of the prototypic sulfated polysaccharide heparin (Figure 2) on in vitro infection. Incubation of Vero cells with heparin (100 μg/mL) 30 min before SARS-CoV injection curtailed infection by 50%. Polyanions have demonstrated antiviral activity (*18*) against enveloped viruses such as HIV and HSV through specific interaction with cells and after inhibition of virus attachment and entry (*14*). Heparin, a prototypic polyanion, inhibits attachment and entry of virus particles into cell by impeding the interaction of the V3 region of gp120 with HIV specific chemokine coreceptor (*19*). In the case of HSV, heparin inhibits the interaction of some HSV envelope glycoprotein to the heparan sulfate that mediates viral attachment to the target cell surface (*20*,*21*). The partial inhibition of SARS-CoV HSR1 by heparin suggests that the envelope proteins coating the SARS-CoV virions might be endowed with positively charged amino acids that could interact with negatively charged sulfate groups present on heparan sulfate proteoglycans expressed on the surface of target cells. Vero cells, the target of both HSV and dengue 4 (*22*) replication, replicate SARS-CoV more efficiently than several other cell lines.

In conclusion, our study characterized a novel SARS-CoV isolate, which was to date the second isolate obtained in Europe. The sequence analysis indicates that SARS-CoV HSR1 clusters together with the Hong Kong, Singapore, and Canada isolates. The coexistence of general coronavirus features with important biologic and molecular specificity of SARS-CoV, together with the evidence that SARS-CoV does not appear to be linked to any of the other three genetic groups of known coronaviruses, strongly suggests it represents a new, fourth genetic coronavirus group, with distinct genomic and biologic features. In light of this evidence, efforts to identify new types of animal coronaviruses as well as to better understand the peculiarities of SARS-CoV are important in addressing its possible animal origin and in understanding the biology of *Coronaviridae* and their evolutionary features.
